# Clinic Report, Veterinary Hospital, University of Pennsylvania

**Published:** 1898-04

**Authors:** S. J. J. Harger, Wm. G. Shaw

**Affiliations:** Professor; Resident Surgeon


### REPORTS OF CASES.

### CLINIC REPORT, VETERINARY HOSPITAL, UNIVERSITY OF PENNSYLVANIA.

S. J. J. Harger, V.M.D., Professor, and Wm. G. Shaw, V.M.D., Resident Surgeon.

Case I. *Median Neurectomy in Ringbone and Tendonitis.*—The patient was a gray draught-gelding, aged. According to the statement of the owner, the animal had been lame for about a month, but judging from the extent of the lesions, the lameness must have existed for a longer period. The symptoms were decided lameness in the off fore-leg, knuckling of the fetlock, and resting the foot on the toe. The local alterations were: Contraction of the perforans and perforatus tendons, which were thickened, indurated, and adherent to each other; local fever had subsided and the inflammatory phenomena were subacute or even chronic. In addition to these symptoms there existed a large ringbone, involving the lower end of the first phalanx, the second, and possibly the articular surface of the os pedis. The corresponding articulations, while not entirely anchylosed, were seriously interfered with mechanically in their movements. The lateral cartilages were ossified.

Median neurectomy was advised and performed after the usual method. All antiseptic precautions were employed, and twenty-four hours before the operation the skin on the inside of the forearm and below the elbow-joint was shaved and cleansed, and a wad of oakum, saturated with a bichloride solution, was applied and maintained with a bandage around the leg and chest. The incision healed nicely without much swelling, no suppuration. Only a very slight lameness remained immediately after the operation. Two weeks after the operation the animal was discharged ready to do his usual work and showed no perceptible lameness.

The reasons for recommending this treatment in preference to any other were evident. The animal was a draught-horse whose value was dependent upon his earnings. The local treatment of ringbones with large exostoses is very unsatisfactory; this as well as firing or blistering the tendons would have required considerable time, without any certainty as to the result. There seems to be no dangerous complication to the neurectomy and no interference subsequently with locomotion. I knew of no other treatment which in such a short time would enable the horse to perform his usual work.

Case II. *Fatty Degeneration of the Kidney in a Cat.*—The subject was a tiger-striped cat, about a year old. The only history obtainable was that for about two weeks she refused to eat and became extremely emaciated. There was a slight discharge from the nose; food was refused. The temperature at the time was only 98.4° F.

The treatment prescribed consisted of tonics and stimulants, such as quinine, nux vomica, and whiskey. On account of the general condition of the animal, tuberculosis was suspected, and .04 c.c. tuberculin was injected. The next morning the temperature was subnormal, and the following day the cat died.

*Autopsy.* Tissues anæmic; catarrhal inflammation of nasal and pharyngeal mucous membranes. The only essential lesion was found in the kidneys. The cortical portion of both kidneys had undergone a complete fatty degeneration; but little normal tissue was left. This was the only lesion that was sufficient to explain the grave general condition, and without any other apparent symptoms liable to be associated with renal trouble.

What the cause of the kidney lesion was could not be determined. It may be said that among others, three principal causes for fatty degeneration of the kidneys are mentioned: 1. Chronic poisoning, the toxic material being constantly excreted by the kidneys, such as arsenic and phosphorus. 2. Continued fever with prolonged congestion of the renal tissues. 3. Pregnancy.

In this particular case, the degeneration being confined exclusively to the part which contains the principle excretory structures, the Malpighian bodies, the first of these three causes may have been in operation, but the exact nature could not be determined, and the history of the case was unsatisfactory. Unfortunately the urine was not examined during life.

Case III. *Polypus of the Superior and Inferior Maxillary Sinuses.*—Aggravated cases of this pathologic condition are not frequently seen, but when they do exist they require either surgical interference, or otherwise render the animal useless. In fact, these cases are more readily heard than seen. Usually no treatment is prescribed. The patient was a bay gelding (draught), aged. History not obtainable. Symptoms: Nostrils dilated; discharge from the right nostril; breathing labored, distressing, and very loud, and of a rasping, snoring-like character, and during rest could be heard at a great distance. Examination of the nasal fossa showed the turbinated bones pushed toward the nasal septum. Percussion revealed dulness over both sinuses. No constitutional symptoms; no local swelling; unable to work.

*Treatment.* The circumstances were such that the removal of the growth was preferred to tracheotomy, and trephining the sinuses was decided upon. A long oval incision was made on the side of the face above the maxillary spine and not infringing upon the attachment of the supermaxillo-labialis muscle. The flap was resected and the two sinuses trephined. Both were completely filled with a growth having a smooth grayish aspect, soft, easily torn and irregular surface, and appearing to be attached principally to the turbinated bones. Unfortunately, no microscopic examination was made. With a pair of bone-forceps the bone was broken away to make a sufficiently large opening to admit the instrument, as well as the fingers, to outline the extent of this growth. The opening was enlarged to suit the circumstances until it was quite large. With a bulldog-forceps, a pair of curved scissors, and drawing-knife, the growth was removed with the greatest care that was possible. The hemorrhage was quite copious. The skin-wound being large, a few sutures were inserted at the upper angle. A portion of the superior turbinated bone was removed with the growth.

The after-treatment was that for an ordinary collection of the sinuses, antiseptic and astringent injections. The wound healed and the discharge ceased. The owner reported htat at the end of a year, when the horse escaped from his observation, there was no recurrence of the disease. Another case was operated on, but we have no information whether the symptoms, which had at last observation disappeared, had subsequently recurred.

CASE IV. *Canker of Both Forefeet.*—Patient, gray gelding, seven years old, was admitted to the free clinic August 11, 1897. The animal was lame in both forefeet. On examining the feet the frogs were found to be covered in their posterior two-thirds with a fungoid growth, which was painful to pressure and covered with a dark-colored, offensive exudate.

*History.* Horse was worked in a milk-wagon. He had been troubled with thrush for almost a year, for which he had been treated by owner and farrier without success.

*Diagnosis.* Pedo-dermatitis, chronic canker of the frogs.

*Treatment.* Shoes were removed and some of the loose horny tissue cut away; animal was very nervous; could do very little without casting him. Placed in creolin soak until following day, softened horn and cleansed feet. August 12th, after preparing instruments, antiseptics, etc., patient was removed from soak and cast. Left front foot was fastened about hock of hind leg in the usual manner. Injected cocaine on plantar nerves. I now cut away the loose particles of frog, and made my first thorough examination. I found that the separation extended quite deeply and behind the upper bars, and almost to the point of the frog, and posterior to the glomes of the heel. Now cut away the bars until no non-adherent material remained. Applied a *tourniquet*. Having the bars cut well away, the frog was quite prominent. With sage-knives I removed the entire frog, cutting well up into the plantar cushion; excess of hemorrhage was checked by thermo-cautery and a 10 per cent. solution of sulphuric acid in alcohol. A flat shoe was now applied and seat of operation dressed with boric and tannic acids, and the ordinary pressure-dressing applied, as well as bandage around the hoof to hold dressing against the heels. Operation on right foot was very similar, only did not remove all the frog. Dressed similar to left, only using more sulphuric acid to check exudation. Patient was now allowed to rise. Subsequent treatment consisted of antiseptics with pressure dressing and occasionally using a cautery. On the 20th of August shoes were applied with movable metal plates and calks; patient was discharged and allowed to do light work, to be returned and have feet dressed. He was returned at stated intervals until November 1st, when feet were thoroughly soaked and shoes with leather soles put on. Frogs sound and healthy.

It may be interesting to note that quite a large ulcer developed on the growing frog of the left foot, which was very difficult to heal, and, after trying various medicinal agents, yielded to a mixture of tannic acid and crude creolin (2 to 1). Animal has been and is now working every day.

